# Supplementary material for: Impact on step count by commitment-based health application
Source: PLoS One. 2024 Aug 12;19(8):e0305765. doi: 10.1371/journal.pone.0305765 (PMC11318894; doi:10.1371/journal.pone.0305765)
Supplement: Attachment — Submitted filename: renamed_5926e.docx [file pone.0305765.s002.docx]

Editor

*PLOS ONE*

March 4, 2024

ID: PONE-D-23-34349

Dear Editor,

On behalf of my co-authors, I would like to express our gratitude to you and interest in our manuscript entitled “Impact on Step Count by Commitment-based Health Application” and for the opportunity to submit a revised version.

We hope you will find the revised version suitable for publication in *PLOS ONE.* We look forward to hearing from you shortly.

Sincerely,

Mira Namba

Keio University School of Medicine

Shinjuku-ku, Shinanomachi 35, Tokyo 1608582, Japan

E-mail: mirrornamba@keio.jp

**Impact on Step Count by Commitment-based Health Application**

**Response Letter**

We would like to thank the Editors and Reviewers for their time and careful consideration of our manuscript. Please find below a detailed description of the revisions and our responses to the editors and reviewers.

**Reviewer 1**

**Comments to the Author**

1) **Line 89: Please elaborate on previous interventions for promoting physical activities and their effectiveness.**
2) **Line 92: It was mentioned that "no studies have been reported on the promotion of physical activity through commitment". As such, whether commitment has been applied in other health-promoting behaviors? What are their effectiveness?**

**Reply**

Thank you for your valuable feedback. We answered 1) and 2) collectively.

We mentioned eating behavior as a commitment to other health behaviors. From the results of a systematic review, we outlined the physical activity promotion interventions and their effects as follows;

**Line 82-92**

Contrary to fewer reports targeting physical activities, a commitment was reported to be most successful when targeting diet [8]. A systematic review on nudges of physical activities shows no commitment study; among 88 studies, 53 were prompting (i.g. prompting taking the stairs through footprints on the floor), 24 were message framing (i.g. emphasizing the benefits of the physical activities), 12 were social comparison (i.g. providing information about others), 8 were feedback (i.g. giving feedback on one’s performance,) 1 was default change (placing a desk at stand-up height) and 1 was anchoring (giving a high-level goal) [9]. However, publication bias might prevent reporting commitment to physical activity.

**Line 241-245**

In the systematic review, the absence of reports on commitment to physical activity is not attributed to publication bias; however, it is considered to be due to the lack of sufficient evidence demonstrating that commitment can effectively promote an increase in the number of steps taken [9].

**Line 264-268**

However, in the presence of the nudges of physical activities, 68% of the studies reported an effect, whereas after removing the intervention, the effect decreased [9]. Not all workplaces can continuously support physical activity interventions for their employees.

3) **Line 102: Please provide references.**

**Reply**

Thank you for your feedback.

Though we saw a Japanese article about “as face-to-face occupational health programs have diminished during the COVID-19 pandemic,” we cannot deny the possibility that face-to-face programs may be increasing after that. Thus, we changed the expression and added the references.

**Line 100-101**

Recently, with the development of remote work, interventions using health applications have been becoming increasingly important [12].

12. Schall MC, Jr., Chen P. Evidence-Based Strategies for Improving Occupational Safety and Health Among Teleworkers During and After the Coronavirus Pandemic. Hum Factors. 2022;64(8):1404-11. Epub 20210108. doi: 10.1177/0018720820984583. PubMed PMID: 33415997; PubMed Central PMCID: PMCPMC9282942.

4) **Line 111: What companies were targeted? What were the inclusion and exclusion criteria of participants?**
5) **Line 115: Please clarify how to allocate the participants into three groups.**

**Reply**

Thank you for your kind comments. We answered 4) and 5) collectively.

We mentioned the target companies, the inclusion and exclusion criteria of participants, and how to allocate the participants into three groups.

**Line 111-123**

Recruitment for the companies was conducted through the Shizuoka Prefectural Government, Japan, and recruitment for the participants was conducted through the companies receiving the letter of participation decision to the program. Allocation was performed based on preferences submitted in advance by each company, and companies that did not submit preferences were assigned randomly. The intervention was conducted in January 2023 [T1], and a one-month follow-up was carried out until February [T2]. The inclusion criteria were regular employees of the company, ≥ 20 years, and the exclusion criteria were those receiving walking-related guidance from medical institutions, and those who failed to submit either of the questionnaires. In the self-commitment and control groups, those using commitment apps were excluded.

**Line 185-187**

Seven companies (five insurance, one finance, and one pharmaceutical sales) participated in the study, and all were allocated according to their previously expressed preferences.

**6) Line 119: Please describe the content of the App.**

**Reply**

Thank you for your comment. We described the content of the App.

**Line 131-137**

Minchalle was designed to form teams of up to five people to achieve behaviors by setting specific goals and recruiting users with the same goal. Daily, participants were recommended to share photos of their goal achievements under their nicknames within their team and provide encouraging comments to other members. In the commitment app group, those who downloaded the app were randomly assigned to teams using a random number table.

**7) Line 133: How to make commitments regarding step count?**

**Reply**

Thank you for your feedback. We added the method of commitment in the self-commitment group.

**Line 139-141**

A kick-off meeting was held in a manner similar to the commitment app group to declare their target number of steps by writing in the chat thread.

**8) Line 166: Please elaborate on the sample size calculation.**

**Reply**

Thank you for your valuable feedback. Referring to a previous study, we described details of the sample size calculation.

**Line 167-171**

Referring to a previous study on promoting behavior by nudges, we assumed an effect size of 0.30, a power of 0.8, and a significant level of 0.05 [15]. A required sample size of 36 in each group was computed by G*Power version 3.1.9.4 (Heinrich-Heine-Universität, Düsseldorf, Germany).

**9) Line 169: What were the outcome measures and how to assess the outcomes?**

**Reply**

Thank you for your valuable feedback. We made the "Outcomes section" and specified the outcomes as "change in number of steps" and "change in 5 health literacy scales" in the section. In the "Statistical Analysis section," we added the details of assessing the outcomes.

**Line 155-157**

2.3.2 Outcomes

Outcomes were changes in step count and health literacy between T1 and T2.

**Line 172-182**

The significance of primary attributes was examined using the Chi-squared test, Fisher's exact test for nominal scale, and the Kruskal-Wallis test for ordinal scale. Concerning outcomes, the change in step count was analyzed using the Kruskal-Wallis test. In addition, other items were divided into two categories, "progress made" and "others." They were analyzed using Chi-squared or Fisher's exact test. Missing values were excluded from the analysis for each item. Data analysis was conducted using SPSS version 28 (IBM Japan, Ltd., Tokyo), and the significance level was set at P < 0.05 (two-sided test). Post hoc testing was corrected using the Bonferroni method (significance level set at P < 0.05/3 = 0.017).

**10) Please add conclusion.**

**Reply**

Thank you for your feedback. We added the conclusion section as follows.

**Line 286-292**

5 Conclusion

Prior research has implied that promoting sustained physical activity through nudges is challenging, and boosting health literacy is essential for the long-term establishment of behaviors. In contrast, this study suggested that commitment-based health applications may increase step count and health literacy, especially due to communication within the app teams through social nudges.
